# A novel method for estrous cycle staging using supervised object detection

**DOI:** 10.1038/s44277-024-00020-x

**Published:** 2025-01-10

**Authors:** Benjamin Babaev, Saachi Goyal, Tushar Arora, Anita Autry, Rachel A. Ross

**Affiliations:** 1 Macaulay Honors College at The City College of New York/CUNY, New York, NY USA; 2 Dominick P. Purpura Department of Neuroscience, Albert Einstein College of Medicine, Bronx, NY USA; 3 Department of Psychiatry and Behavioral Sciences, Albert Einstein College of Medicine, Bronx, NY USA

**Keywords:** Machine learning, Reproductive biology

## Abstract

The estrous cycle regulates reproductive events and hormone changes in female mammals and is analogous to the menstrual cycle in humans. Monitoring this cycle is necessary as it serves as a biomarker for overall health and is crucial for interpreting study results. The estrous cycle comprises four stages influenced by fluctuating levels of hormones, mainly estradiol and progesterone. Tracking the cycle traditionally relies on vaginal cytology, which categorizes stages based on three epithelial cell concentrations. However, this method has limitations, including time-consuming training and variability among researchers. This study assesses the feasibility and reliability of two previous image classification models as well as introducing an alternative method of machine learning to address the challenges posed by manual vaginal cytology and image classification. An object detection-based machine learning model, Object Detection Estrous Staging (ODES), was employed to identify cell types throughout the estrous cycle in mice. A dataset of 730 vaginal cytology images with four different stains was used, with 335 images for training, 45 for validation, and 350 for testing. A novel, accurate set of rules for classification was derived by analyzing training images. ODES achieved an average accuracy of 80% in classifying cycle stages, comparable to human accuracy (66%) and previous image classification models (41–79%). The efficiency of ODES, processing 100 test images in just 2.67 minutes, makes it a valuable tool for large-scale neuropsychiatric studies involving female rodents and also encourages the integration of this variable into neurological and psychiatric research. These results demonstrate that ODES offers a fast, reliable, and accessible method for estrous cycle monitoring, potentially improving how researchers approach sex-based variables in neuropsychiatric studies.

## Lay Summary

This study presents a new machine learning tool called ODES (Object Detection for Estrous Staging) to determine the estrogen-related cycle stage in female mice. Unlike traditional models that classify images, ODES detects and classifies cell types, achieving 80% accuracy; comparable to human performance and previous models. It processes images quickly, making it ideal for large studies. By efficiently tracking the estrous cycle, ODES improves how researchers consider sex-based differences in neuropsychiatric research.

## Introduction

### The estrous cycle

The estrous cycle, analogous to the human menstrual cycle, is the reproductive cycle of female non-primate vertebrates including mice and rats. The estrous cycle is divided into 4 stages: diestrus, proestrus, estrus, and metestrus. These stages are influenced by fluctuating levels of ovarian hormones, notably estradiol and progesterone. The cycle influences brain activity [1] and physiological characteristics, such as changes in hypothalamic-pituitary-adrenal axis activity [2], feeding, and energy metabolism [3]. However, the effects of the cycle are often overlooked in neuroscience investigations and psychiatric care [1]. Historically, research has predominantly utilized male subjects, resulting in an insufficient understanding of female disease pathology and suboptimal treatment plans for females [4]. The common misconception that data/results from female studies are more variable than groups of males has not been proven true. Instead, it seems that there are fundamental differences as a result of sex-based variation [5], including some that are due to hormone fluctuations across the cycle. Characterizing the stages of the estrous cycle serves as a crucial tool for understanding the female biological context [6]. Incorporating the estrous cycle in studies related to rodent behavior and genetic interactions can improve the quality of neuroscience findings and more accurately identify hormone-fluctuation-related sex differences. Recognizing hormone variance is crucial in examining behavior, pharmacology, and neurological changes, and drawing parallels between animals and humans provides an opportunity to understand the mechanisms that underlie these changes.

## Estrous stage classification techniques

**Fig. 1:**
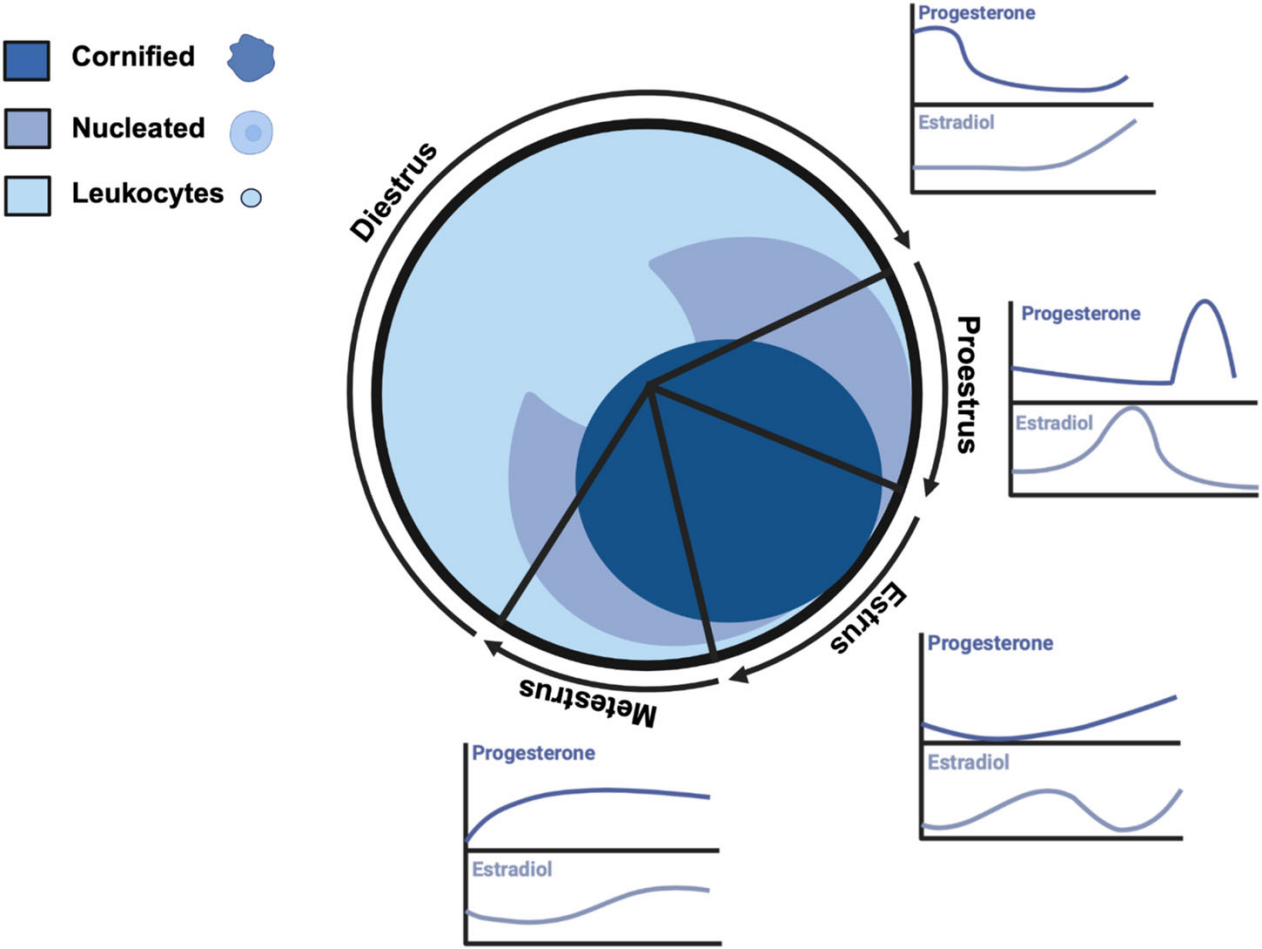
Cell Populations and Hormone Levels Throughout the Estrous Cycle. Cell populations and relative sex hormone levels throughout the estrous cycle in female mice (Adapted from Byers et al. [20] and Donner & Lowry [6]). Schematic made with BioRender.

The identification of the estrous stage is typically done using vaginal cytology, visual assessment of the vagina, or vaginal wall impedance [7]. Vaginal cytology is the most common indirect tracking method and involves microscopic examination of vaginal smears, analyzing the shape, size, and number of cells to determine each stage. Epithelial cells are found in the vaginal lining and change in appearance throughout the estrous cycle. Cornified cells, which lack a clear nucleus, are most abundant during estrus which lasts 12–48 hours. Nucleated epithelial cells are present in higher numbers during proestrus which lasts less than 24 hours. Leukocytes, or white blood cells, are most abundant during diestrus which lasts 48–72 hours. The metestrus stage shows a mixed cell population with no distinct majority (Fig. 1) and lasts 8–24 hours. Because the hormone ratios are similar and it becomes difficult to differentiate by cytology, metestrus is also commonly combined with diestrus, or referred to as early diestrus.

## Drawbacks of vaginal cytology and previously used machine learning strategies

Vaginal cytology has limitations: it requires significant time and training to ensure proper classification, and there can be variability in classification among researchers. To overcome these drawbacks, previous research has explored the application of image classification. These supervised machine-learning techniques utilize convolutional neural networks which have proven to be more accurate and efficient than human experts at diagnosing various medical disorders like cancer, heart disease, and neurological conditions based on classification of morphological features in images and signal analysis [8]. The ability to do so arises from analyzing vast amounts of data, which allows for the identification of patterns and anomalies that may be overlooked by human specialists. However, a limitation of image classification is the inability to identify the location of various objects within an image, therefore the exact rationale (which image features are used to determine classification) behind the model’s stage classifications is unknown [9].

Two supervised image classification models for vaginal smears have been previously described. The SECREIT model [10] was developed to identify images representing three stages of the estrous cycle—diestrus, estrus, and proestrus—using solely Giemsa stain, achieving a reported accuracy of 93%. The EstrousNet Model [11] was trained on thousands of images from various labs, encompassing different animal species (mice and rats), stains, and magnifications, with a reported accuracy of 83%. However, it is yet unknown whether the accuracies of these models are reproducible on new data sets.

## Object detection

Supervised learning is a common form of machine learning that can create models to accurately predict outcomes for new, previously unseen data when trained on large labeled datasets [12]. It is widely used for tasks such as image classification and object detection. Image classification involves assigning a single label to an entire image. Object detection, on the other hand, involves the identification and localization of objects in a given image. This technique is useful for its ability to detect objects across different classes, sizes, orientations, and scales [13]. Its advantages over image classification are that it recognizes both the presence and position of various objects, each with a respective label. Additionally, it has the capacity to combine information from an array of verified objects in order to classify the image. It requires human input to define individual objects, which can be a source of bias, but this is mitigated in the context of a limited object dataset such as cells seen in vaginal smears.

YOLOv8, short for “You Only Look Once” version 8, is a freely available object detection model that uses the principles of convolutional neural networks to perform real-time object detection with high precision. We used the YOLOv8 model to achieve generalizability and reliability in classifying vaginal cytology images, demonstrating performance comparable to both previously reported image classification models and human experts. This standardized, rule-based approach minimizes variability among researchers and reduces classification time. Here we demonstrate the potential of a supervised object detection paradigm called Object Detection Estrous Staging (ODES) in automating cell type classification and cell counting, offering a method similar to human expert classification of vaginal cytology, with enhanced efficiency and generalizability.

## Methods

### Dataset and preparation

The dataset for this study was composed of mouse vaginal cytology images sourced from the Ross Lab, Verstegen Lab, Correa Lab, Sano Lab, and EstrousBank, a free online resource with images from five different labs [11]. The dataset staining techniques included the Giemsa stain, Crystal Violet, Shorr stain, and H&E stain, and multiple magnification levels. The data was divided into three sets of randomly chosen images: 335 images for training, 45 for validation, and 350 unseen images for testing.

### Image annotation

**Fig. 2:**
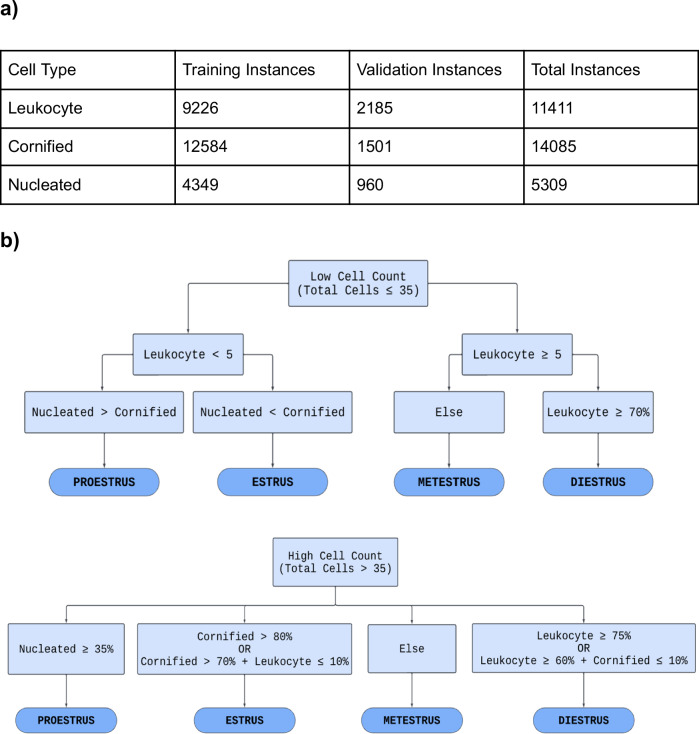
Overview of Dataset and Estrous Stage Classification. **a** Table displaying the distribution of cell types across the training and validation datasets. The leukocyte class consisted of 9,226 instances in the training set and 2,185 in the validation set, with a total of 11,411 instances. The cornified class consisted of 12,584 instances in the training dataset and 1,501 in the validation dataset, with a total of 14,085 instances. The nucleated class had 4,349 instances in the training set and 960 instances in the validation set, resulting in a total of 5,309 instances. Each cell training instance was generated by the manual labeling process. **b** Flowchart that is a visual representation of the algorithm (written in Python) used to determine the estrous stage by assessing the cell count. It divides the process into two algorithms: one for low cell count and another for high cell count. Through a series of binary decisions based on the criteria provided in each block, the model narrows down the classification to one of the four estrous stages.

To prepare the dataset for training, 335 images were annotated using a freely available online labeling tool makesense.ai. Each image in the training set was manually annotated by drawing rectangular bounding boxes around each cell and tagging the cell with its corresponding label: “Leukocyte”, “Cornified”, or “Nucleated”. The training set had 9226 instances of leukocytes, 12,584 instances of cornified cells, and 4349 instances of nucleated cells (Fig. 2a).

### Exporting annotated images

Post-labeling, the annotations of the images were exported as .txt files. We used a class index for each cell type: 0 for Leukocyte, 1 for Cornified, and 2 for Nucleated. Additionally, the annotation included four unique values to represent the x-center coordinate, y-center coordinate, and width and height of the bounding box [14]. The raw images were downloaded into the image folder and the corresponding YOLO text document, with the same name as their corresponding image, into the labels folder. The images with their corresponding .txt files were sorted randomly into training and validation folders.

### YOLOv8 architecture and features

The backbone of the YOLOv8 object detection framework is an advanced version of the CSPDarknet53 backbone, which reduces processing time and memory usage [15, 16]. CSPDarknet53 is an advanced neural network architecture that focuses on efficient feature extraction from input images [17]. It uses a technique called the Cross Stage Partial (CSP) design to make sure the model is fast and detailed, helping it better recognize important visual elements and the overall image [18, 19].

The model includes a Spatial Pyramid Pooling (SPPF) layer to improve the likelihood of correct output and speed of object detection by optimizing feature extraction [16]. Unlike previous methods which divide input images into regions of various sizes, SPPF processes the image sequentially using “same-sized analysis windows” (kernels) multiple times [19]. This approach allows the model to capture details at multiple scales, which is important for object detection [19]. By processing the image in this sequential manner, SPPF achieves better performance without sacrificing accuracy [19]. The model also includes features like spatial attention, which improves the model’s ability to identify objects in complex backgrounds [16].

### Model training

The pre-trained YOLOv8s model (a small version of YOLOv8) was utilized within a Google Colaboratory environment, which provided access to the Tesla T4 GPU. The model was adapted to recognize specific classes of cells (leukocytes, cornified cells, nucleated cells) not included in its original training set. This was achieved by employing a custom dataset configuration to guide the model’s training process. The document “customdata.yaml” provided explicit paths to the training and validation sets, and introduced the three cell-type classes with their respective class indices. Images of various sizes were used for training and validation but for YOLOv8, the image-size parameter was set to 640. The model was trained for 62 epochs, during which various augmentations were applied to diversify and enlarge the training set. The default augmentations utilized by YOLOv8 were applied, including changes in hue, saturation, brightness, translation, scaling, horizontal flipping, mosaic augmentation, random color and rotation variations, and random erasing. The maximum object detection parameter was set to 1000. The batch size, which is the number of samples processed per training iteration, was set to 18. The patience was set to 20 epochs to prevent overfitting.

### Rule-based classification

A preliminary rule-based stage classification system (Fig. 2), written in Python, was developed using a qualitative diagram of the estrous cycle [20]. Ground truth for the rules was determined by analyzing the average number of each cell type present within an image for a given stage. This data was obtained from the initial labeling of cells within the training images. Ground truth for cell identification was determined based on the distinct characteristics of each cell type: Cornified cells lack a clear nucleus. Nucleated cells have a defined nucleus. Leukocytes are much smaller and round. The performance of the trained model was evaluated by observing its outputs when presented with novel test images it had not previously encountered. All images in the test set were initially labeled by EstrousBank and subsequently reviewed by three experts. For an image to be included, at least two of the three reviewers needed to agree with the label; images marked as incorrect by at least two experts were excluded. This process established the test set as the ground truth.

The rules and training criteria were refined through a feedback loop to improve the model’s accuracy and reliability to better represent the cell populations of each stage. The feedback loop involved training the model on the training set, evaluating the model’s performance on the validation set, and adjusting either the rules or training criteria based on validation results (Fig. 2a). If the model fell short in cell classification, the validation image was annotated, placed into the training set, and retrained. If the model misclassified the estrous stage and the cell classification was accurate, the rule-based stage classification system was adjusted (Supplemental Fig. 1). An additional set of rules was developed to account for cases with low cell counts (Fig. 2b). For instances where ODES output showed a lower cell count than expected or a higher background count a “Biological impossibility tag” was implemented. This tag flags scenarios where the cell counts are low, which could indicate that the model failed to detect clusters of cells and instead misclassified them as background. The tag is indicated by three asterisks (***) in the output classification table the user receives upon running ODES on a set of vaginal cytology images. Once the rules and weights were finalized, the model was complete.

### Model comparison

The performance of ODES was tested against human accuracy. The study protocol was reviewed by the Albert Einstein College of Medicine Institutional Review Board. Four human researchers were timed while classifying a set of 100 images. The model ran through the same set of 100 images and was timed. The test set contained 25 random images for each stage obtained from EstrousBank and SECREIT data that ODES had not seen before.

The model was also compared with two previously published supervised image classification machine learning models: EstrousNet and SECREIT. One test set was utilized consisting of 200 images sourced from EstrousBank data, with 50 images representing each of the four estrous stages, and another consisting of 150 images from SECREIT data, with the same stage distribution but excluding the metestrus stage, as their data combined diestrus and metestrus. Each image was unique with no duplicates in the test set, and none were used in the training or validation sets.

The accuracy of the participants and the model was determined by comparing their classifications to the established benchmarks provided by EstrousBank and SECREIT datasets, which were considered the correct stages for this experiment. However, it is important to distinguish between accuracy and reliability in this context. Reliability refers to the correctness of each individual image classification. Accuracy, on the other hand, is the consistency of estrous stage labeling across the dataset which describes the model’s classification performance compared to the ground truth.

### Vaginal cytology preparation and ODES setup

Regardless of manual classification or using machine learning, vaginal cytology preparation remains the same. This process includes pipetting fluid in and out of the mouse vaginal opening, placing the fluid onto a slide allowing it to dry which takes about 15–30 minutes depending on how much water was used. Staining then takes about 15–30 minutes depending on how many slides there are. Then the slides must air dry completely before imaging which can take over an hour, regardless of whether staining was performed or not. In total, the preparation process can take 2–3 hours.

To set up ODES, the code and weights files are downloaded from GitHub and a new environment needs to be created with the required packages installed. The initial setup of the environment for ODES takes about 5 minutes and only needs to be done once. Once the environment is ready, running the model takes less than a minute. The researcher provides the path to the images directory and executes ODES, a process that takes less than a minute. We now include a brief YouTube tutorial on GitHub to assist people in running the code.

### Statistics

A nested one-way ANOVA followed by Tukey’s multiple comparisons test was used to compare the classification accuracy of ODES with EstrousNet and SECREIT across estrous cycle stages. Cohen’s d was used to compare the effect size of the accuracy difference between ODES, EstrousNet, and SECREIT. A two-way ANOVA followed by Sidák’s multiple comparisons test was used to compare classification time per image and stage classification accuracy between ODES and human experts across estrous cycle stages.

## Results

### ODES cell classification

**Fig. 3:**
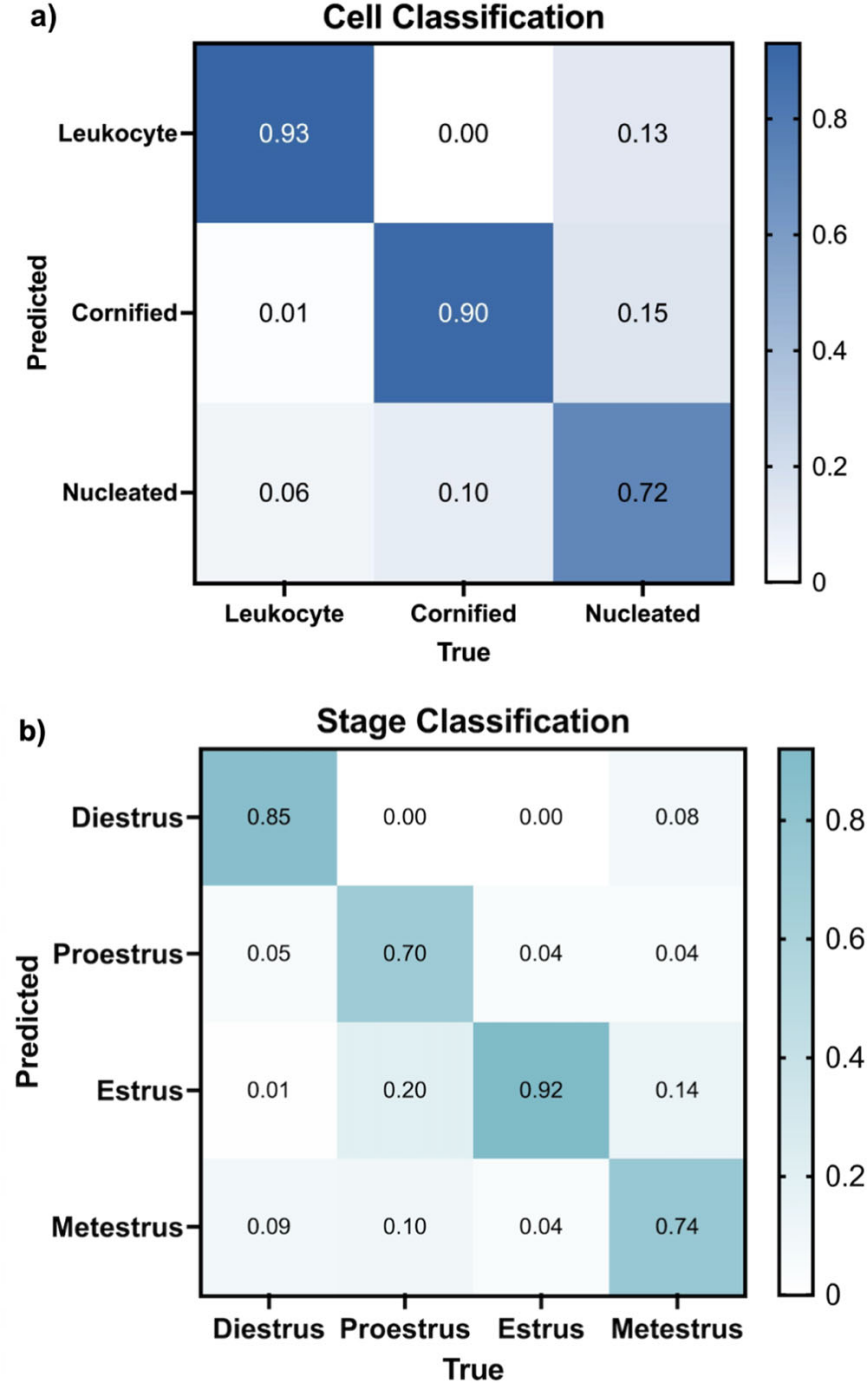
Normalized Confusion Matrices for ODES Performance. **a** Normalized confusion matrix that evaluates the cell classification performance of ODES on the validation set. The x-axis represents the true cell type (Leukocyte, Cornified, Nucleated), and the y-axis shows the predicted labels. The diagonal elements of the matrix represent the instances that were correctly classified by the model, while the other elements represent the number of instances that were misclassified. **b** Normalized confusion matrix for ODES stage classification of 350 test images (100 diestrus, 100 proestrus, 100 estrus, 50 metestrus). The x-axis indicates the true stages (Diestrus, Proestrus, Estrus, Metestrus), while the y-axis shows the predicted stages. Similar to Fig. 3a, the diagonal represents the correct predictions for each stage and the other cells represent the incorrect predictions.

The trained model’s cell classification ability was analyzed on the 45-image validation set. The performance is demonstrated with a confusion matrix, providing the likelihood of correctly predicting each cell type: leukocyte, cornified, and nucleated (Fig. 3a). Leukocytes were correctly identified in 93% of cases with only a 6% misclassification rate with nucleated cells and 1% as cornified cells. Cornified cell identification was 90% accurate, with 10% of instances being mistaken for nucleated cells. The predictive probability for nucleated cells was 72%, with misclassification as cornified cells to 15% and leukocytes at 13%. In this classification, many cells, particularly those that overlap and clump together, were mistaken for background (Supplemental Fig. 2). This was primarily the case for leukocytes which tend to be clustered, posing challenges for ODES in distinguishing them from background elements. To mitigate this issue, a ‘Biological Impossibility Tag’ was introduced to flag low cell counts that may suggest the presence of uncounted cell clusters or other anomalies. It is recommended that ODES users review the images with low cell counts. Among 350 images in the test set, 19 images were tagged (~5% of images).

### ODES stage classification

Using the classification rules (Fig. 2b) and trained weights derived from cell classification training, ODES classified the stages of the estrous cycle in a test set comprising 350 previously unseen images (200 from EstrousBank, 150 from SECREIT). The stage classification confusion matrix demonstrates the accuracy of the model for each estrous stage (Fig. 3b). In the diestrus stage, ODES achieved an accuracy of 85% with misclassifications 9% of the time as metestrus, 1% as estrus, and 5% as proestrus. The proestrus stage was correctly predicted with an accuracy of 70%, though 20% of instances were misclassified as estrus and 10% as metestrus. The estrus stage had an accuracy of 92%, where 4% of instances were misclassified as proestrus and metestrus each. The metestrus stage had 74% correct classification with misclassification as estrus in 14% of instances, proestrus in 4%, estrus in 14%, and diestrus in the remaining 8% of instances.

To test if any stain provided greater accuracy than others, we evaluated outcomes for each stain type separately. The test set of 350 images was used to test stain accuracy out of which 179 had the Giemsa stain (29 from EstrousBank and 150 from SECREIT), 42 had the H&E stain, and 129 had the Shorr stain. ODES achieved an accuracy of 87% for Giemsa, 79% for H&E, and 75% for Shorr stain. The trend toward best performance on images with a Giemsa stain was not statistically significant (Supplemental Fig. 3b). There was no significant difference in ODES’s accuracy regarding stain across all stages. Though not statistically significant, proestrus demonstrates a higher accuracy with the Giemsa stain compared to the H&E and Shorr stains. (Supplemental Fig. 3c).

### Comparison of ODES with image classification models

**Fig. 4:**
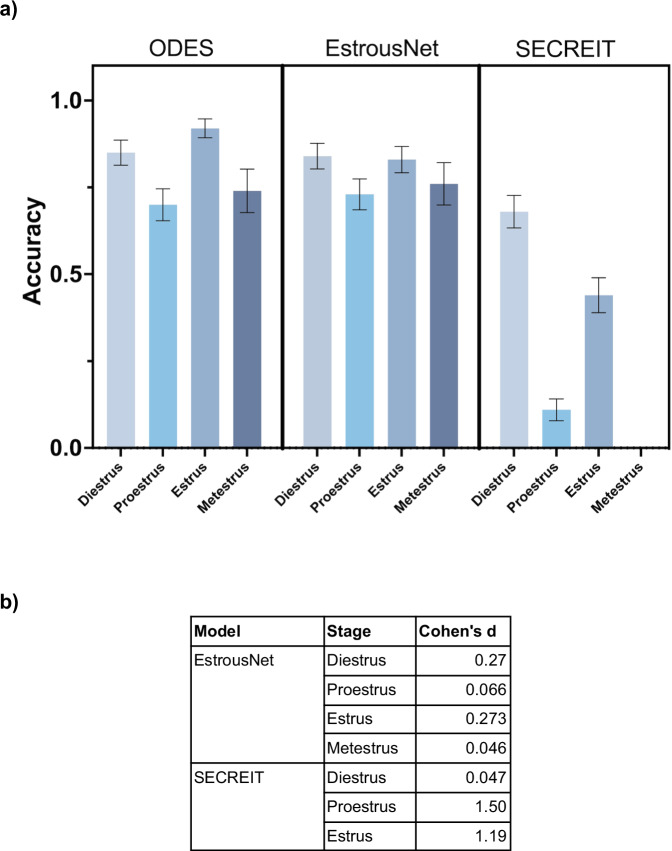
Classification Accuracy Comparison and Effect Size across models. **a** Classification accuracy of ODES compared to image classification ML models EstrousNet and SECREIT across the four estrous cycle stages. SECREIT was not trained to classify the metestrus stage, therefore data for SECREIT metestrus is not available. A nested one-way ANOVA revealed significant differences in model performance across the estrous stages (*p* < 0.05). Tukey’s multiple comparisons test indicated no significant difference between the performance of ODES and EstrousNet but there is a significant difference between ODES and SECREIT (*p* < 0.03). **b** Table comparing the effect sizes of ODES to EstrousNet and SECREIT on the accuracy of estrous stage classification using Cohen’s d.

The stage classification accuracy of ODES was compared to two supervised image classification machine learning models, EstrousNet and SECREIT, across the four stages of the estrous cycle (Fig. 4a). The test set contained randomly chosen images for each estrous stage obtained from EstrousBank (200 images, 4 stages) and SECREIT data (150 images, 3 stages). For the diestrus stage, ODES achieved an average classification accuracy of 85%. In contrast, EstrousNet and SECREIT had comparable accuracies of 84% and 68%, respectively. In the proestrus stage, ODES’s accuracy averaged 70%, while EstrousNet classified with 73% accuracy, and SECREIT with 11%. During the estrus stage, ODES had an accuracy of 92%, with EstrousNet at 83% and SECREIT at 44%. For the metestrus stage, ODES had an average accuracy of 74%, and EstrousNet’s accuracy was 76%. SECREIT’s performance in the metestrus stage was not assessed as it was not trained to classify metestrus. Taken together, ODES shows comparable accuracy and generalizability compared to other image classification models for vaginal cytology stage classification.

In assessing the performance of the ODES model compared to EstrousNet and SECREIT across various estrous phases, Cohen’s d values offer a quantitative measure of the effect size. This effect size analysis reveals that ODES outperforms SECREIT for proestrus and estrus but is comparable to EstrousNet across all stages (Fig. 4b). Against SECREIT, the effect size is minimal for diestrus at Cohen’s d of 0.047. In the proestrus phase, ODES performs better with Cohen’s d of 1.50 compared to SECREIT. In the estrus phase, ODES has an effect size of 1.10 against SECREIT. This quantitative analysis suggests that ODES is a reliable and effective model demonstrating comparable accuracy to EstrousNet across all stages and outperforming SECREIT in proestrus and estrus.

### Comparison of ODES to Human Experts

**Fig. 5:**
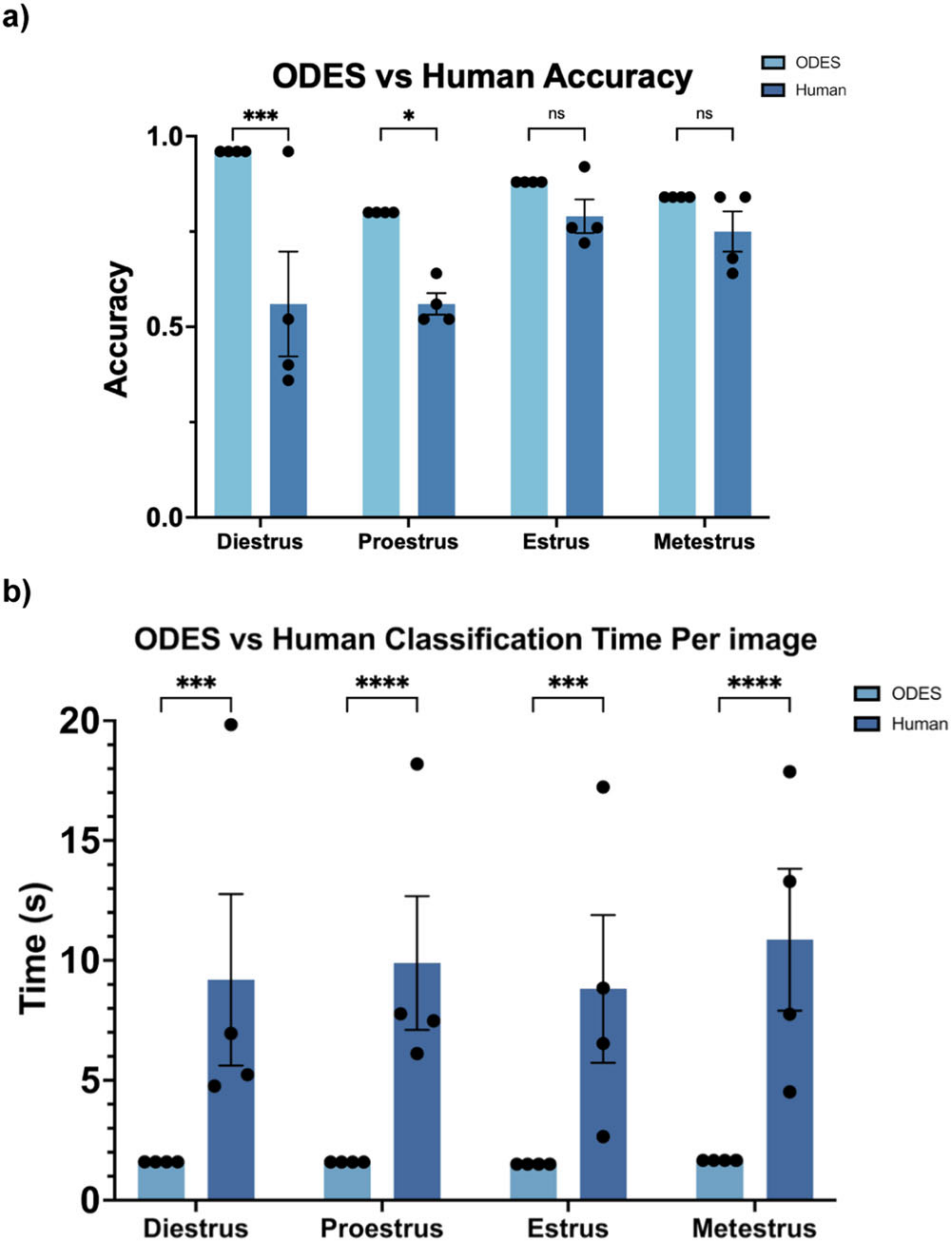
Accuracy and Speed Comparison Between ODES and Human Experts. **a** Comparison of ODES and human accuracy in estrous cycle stages classification. The error bars indicate the SEM, while individual data points represent different trials. ODES was run four times to statistically compare to four human trials. In a two-way ANOVA, there was a significant difference between ODES and Human accuracy (*p* < 0.0001). Using Sidák’s multiple comparisons test, significant differences were present for diestrus and proestrus, with *p*-values of 0.0010 and 0.0384 respectively, however, there were no significant differences in estrus and metestrus, both with *p*-values of 0.7195. **b** Comparison of ODES and human classification time per image in estrous cycle stages classification. The error bars indicate the SEM. The individual data points represent the time per image averaged over 25 images. In a two-way ANOVA, there was a significant difference between ODES and Human time (*p* < 0.001). Using Sidák’s multiple comparisons test, significant differences were present for all four stages (*p* < 0.0015).

The accuracy rates for both ODES and human classifiers were compared across the four estrus cycle stages (Fig. 5a). ODES achieved high accuracy rates with averages of 96% for diestrus, 80% for proestrus, 88% for estrus, and 84% for metestrus. In comparison, human accuracy rates were measurably but not significantly lower, with an average of 56% for both diestrus and proestrus, 79% for estrus, and 75% for metestrus. The average classification time per image was also compared between human experts and ODES (Fig. 5b). The human experts averaged 9.7 seconds per image, while ODES reduced the classification time to an average of 1.6 seconds per image (*p* < 0.001). Thus, ODES preserves the accuracy and enhances the efficiency of vaginal cytology-based estrous cycle staging compared to human experts.

## Discussion

We used object detection to train a machine-learning model to approach images of vaginal cytology like an expert scientist. We trained the model to recognize individual cell types and created a rule scheme that allowed the model to define the estrous cycle stage based on the relative percentage of each cell type seen in each image. ODES demonstrates its classification capabilities by matching image classification models in generalizability and reliability and surpassing human experts in classification speed. It has also shown consistent accuracy and reliability across various stains, magnifications, and data sources. The consistent performance of ODES in classifying estrous stages may be attributed to its novel approach that integrates object detection with subsequent classification based on detected cell types. This methodology provides a contrast to traditional image classification models that rely only on supervised learning paradigms [10, 11]. Previous models typically label entire images according to the estrous stage, without the ability to identify individual cell types [10, 11]. This may have led to pattern detections within the image that do not capture the features essential for accurate stage classification.

By identifying specific cell types and quantifying their proportions, ODES’s approach allows for a more detailed and biologically relevant analysis. There is a well-defined, limited data set that includes all possible cell types that would be present in a given vaginal cytology slide. Therefore, it is reasonably simple to train the paradigm to recognize all objects in this limited set, and there is minimal bias imposed by human input. The model’s ability to match cellular features to their specific types and then classify them based on these more detailed metrics may have ensured a high degree of accuracy in stage classification, even with confusion between some cell types and the background. This two-step method allows the model to be generalized and scalable in ways that less supervised, or less detail-oriented models may not.

While the goal of machine learning is generally to reduce human input, it seems that in this case, there is a significant benefit to some brief human involvement in the supervised learning process. The model’s predicted results are compared to data provided by humans, and this comparison is used to calculate the loss function, which measures how well the model performs [21, 22]. This ensures that the model is aligned with patterns recognized by human experts, rather than forming potentially incorrect assumptions based on purely algorithmic interpretations. The goal is to make this loss as small as possible by adjusting the model’s parameters to minimize the difference between predicted and actual labels. This is important in cell classification where sample variability can be high but remains advantageous because the set of objects is limited by biology. Future work should evaluate whether ODES reliability is maintained in a new context, such as a different stain type, or a dataset from another animal model.

### Challenges with specific cell types and stages

All models, including ODES, had the lowest accuracy of stage classification for the proestrus stage. Similarly, the likelihood that ODES correctly identifies nucleated cells, which are the predominant cell type during proestrus, was the lowest for all cell types. There are a couple of reasons for this poor performance. First, proestrus staging is dependent on only one cell type, unlike the other stages. Second, the limited duration of the proestrus stage, typically less than 24 hours, led to a smaller dataset for this phase. Smaller data sets often lead to imbalanced data and model overfitting or underfitting due to inappropriate feature dimensions, a common challenge in machine learning [23]. The limited data availability therefore restricts ODES’s ability to accurately classify nucleated cells, affecting the overall classification performance for the proestrus stage. Additionally, certain stains appear to be more effective at highlighting specific cell characteristics, which impacts image classification. For example, it seems the Giemsa stain defines the nuclei of nucleated cells better than other stains, leading to improved accuracy for proestrus staging (Supplemental Fig. 3c). To address these factors, a redefined threshold for proestrus classification is suggested in the stage classification flow chart: a nucleated cell percentage of 35% or higher, instead of a simple majority as initially encoded based on Byers et al. [20]. This improved the accuracy of the ODES proestrus readout.

### Limitations of ODES

ODES does not perform well on unstained images, which could be seen as a limitation as this adds an extra step for human researchers. The lack of staining makes it more difficult for the model to distinguish the individual cell based on its boundaries and characteristics. However, since staining images is the standard procedure for estrous staging, this limitation is mitigated [24]. Additionally, ODES has difficulties classifying individual cells when they are clumped together, and classifying stages when cell count is very low. The difficulties with clumped cells arise due to the lack of clear boundaries between the cells. Cells can overlap each other, creating complex morphological features that are challenging to distinguish. Since ODES was trained to make decisions similar to human judgment, it encounters the same problems with clumped cells that humans do. This issue can also be compounded by biological and histological constraints, such as sticky vaginal excretions that lead to cell clumping, making the smear method less effective for consistent daily analysis across all animals. Furthermore, ODES’s cell classifications may be affected by variations in stain color and intensity, as well as lighting conditions during image capture (such as brightfield microscopy), as these factors can introduce inconsistencies in color perception and contrast.

Additionally, since the model’s training involves human input, any bias or inconsistency in cell annotations can directly influence the model’s learning patterns [25]. For instance, if the annotations provided by human experts vary due to subjective interpretations of what constitutes a particular cell type or boundary, the model will learn these inconsistencies, potentially reducing its reliability and generalizability. Additionally, human annotators can have varying levels of confidence and criteria in classification [25]. Variability can lead to a model that is uncertain or inconsistent in its classifications, mirroring the inconsistencies of its training data.

Finally, while it is common practice to classify estrous images by considering their order [20], ODES currently does not incorporate data in a particular sequence. Integrating this logic, potentially by standardizing the order of the dataset, could improve accuracy outcomes, especially across data collected over time from individual animals.

### Insights from supervised classification at different levels

ODES’s high accuracy in stage classification compared to cell classification offers valuable insights into stage determination by the model. It seems that the primary requirement of stage classification accuracy is to correctly identify the dominant cell type within the image a sufficient number of times, rather than achieving perfect predictive probability for every cell. As a result, misclassifications of individual cells have a reduced impact. If the model can accurately classify a majority of cells correctly, the true stage determination is likely to be achieved. This principle is seen in other studies as well. For example, a study using Phase Imaging with Computational Specificity—a novel imaging technique that combines label-free cellular imaging with neural networks—has shown that even with large sample sizes and varying cell counts, accurate cell stage classification is achievable [26, 27]. The study determined cell stages by analyzing cell body masks and counting fluorescence signals, taking the majority label as the final classification. This reinforces the idea that classifying the majority of cells correctly can be sufficient for accurate results, suggesting that ODES is likely to be similarly scalable.

The cell classification confusion matrix with background (Supplemental Fig. 2) shows that most of the error in cell classification occurs when the cells are mistaken for background. Upon review of these images, we found this is due to challenges in recognizing clusters of cells. The difficulty seems to lie in ODES’s ability to recognize and differentiate the boundaries of overlapping individual cells. In these instances, ODES output showed a lower cell count than expected or a higher background count, both of which led to the implementation of the “Biological impossibility tag”, which alerts users that the image may be misclassified. By flagging potential errors, we introduce a secondary review process: both manual check by a human expert and classification through another automated rule set designed to classify images with low cell count. This approach enhances the overall accuracy and reliability of the system.

### Future Directions for ODES

Future improvements for ODES focus on two main areas: expanding the dataset and upgrading the model architecture. The inclusion of unstained images into the training and validation set, and ordered images defined according to individual animals may enhance the model’s adaptability to various imaging conditions as well as further improve its generalizability.

The training of ODES was limited in computational resources, such as storage capacity in the training environment, which restricted the ability to employ larger, more complex YOLO models. With access to hardware with greater GPU capacity, it would be feasible to implement larger and more complex versions of the YOLOv8 model. Additionally, with the recent release of YOLOv9, there is potential for improved training results when compared to YOLOv8. This is attributed to its innovative approach to mitigating information loss challenges commonly observed in deep neural networks.

Additionally, in assessing the performance of ODES, it is important to consider the data used, particularly the stage labels provided by various labs. There is a possibility that these labels may not have been completely accurate. This potential mislabeling in the source data can affect the reliability and accuracy of the model by introducing a source of error that is external to the model’s logic and training process. Although human experts reviewed each test image and relied on a majority decision for labeling, this process may still have inconsistencies. This approach, however, is relevant to realistic lab practices where expert classifications may vary. Nevertheless, because the process included multiple expert reviews and checks, there is greater confidence in the overall reliability of the test labels, making the model more robust.

### Maintenance and reliability in open source (estrous) models

Open source models on platforms like GitHub require regular updates to provide ongoing functionality for the user. Many of these models depend on external software libraries that may be updated to include new features and bug fixes. If the model code is not updated to match these changes, it can lead to issues with compatibility and reduce the model’s ability to function as expected. Therefore, routine maintenance and updates to open-source models help maintain optimal performance and consistent results. Additionally, updates to the backend code, including changes to the model’s architecture or algorithms, can impact performance. In some cases, these updates may improve model speed or accuracy, but they can also introduce variability in performance or even cause unexpected outcomes. Therefore, keeping these models up to date is crucial for maintaining their reliability in estrous staging.

## Conclusions

ODES has demonstrated remarkable generalizability and accuracy in classifying vaginal cytology images, standing as an equal contender to both image classification models and human experts. The ability to easily implement ODES on a laptop makes it accessible to a wide range of researchers, regardless of their computational resources or expertise. Its standardized, rule-based approach can reduce variability among researchers and speed up the classification process. ODES enables researchers to quickly and efficiently determine the estrous stage of female mice at multiple time points. This is particularly valuable in studies that require precise tracking of the estrous cycle in female mice and can lead to improved diagnostic precision in clinical settings for women. Despite its strengths, ODES faces challenges such as difficulty with unstained images and classification in clustered cell scenarios. These limitations can be mitigated with the expansion of the training dataset to incorporate unstained images and the shift to a newer or more complex model architecture.

## Supplementary information

Supplementary information

## Data Availability

The data that support the findings of this study are openly available in the Ross Lab Github repository: https://github.com/rrosslab/ODES_Object_Detection_For_Estrous_Staging/tree/main/Train%20your%20own%20model%20(OPTIONAL)/traindata/images and in the EstrousBank BioImage Archive https://www.ebi.ac.uk/biostudies/bioimages/studies/S-BIAD545 associated with the unique identifier 10.1038/s41598-022-22392-w.
