# Supplementary material for: A novel method for estrous cycle staging using supervised object detection
Source: NPP Digit Psychiatry Neurosci. 2025 Jan 10;3:3. doi: 10.1038/s44277-024-00020-x (PMC12510444; doi:10.1038/s44277-024-00020-x)
Supplement: Supplementary file 1 — Supplementary information [file 44277_2024_20_MOESM1_ESM.pdf]

Supplementary Figures

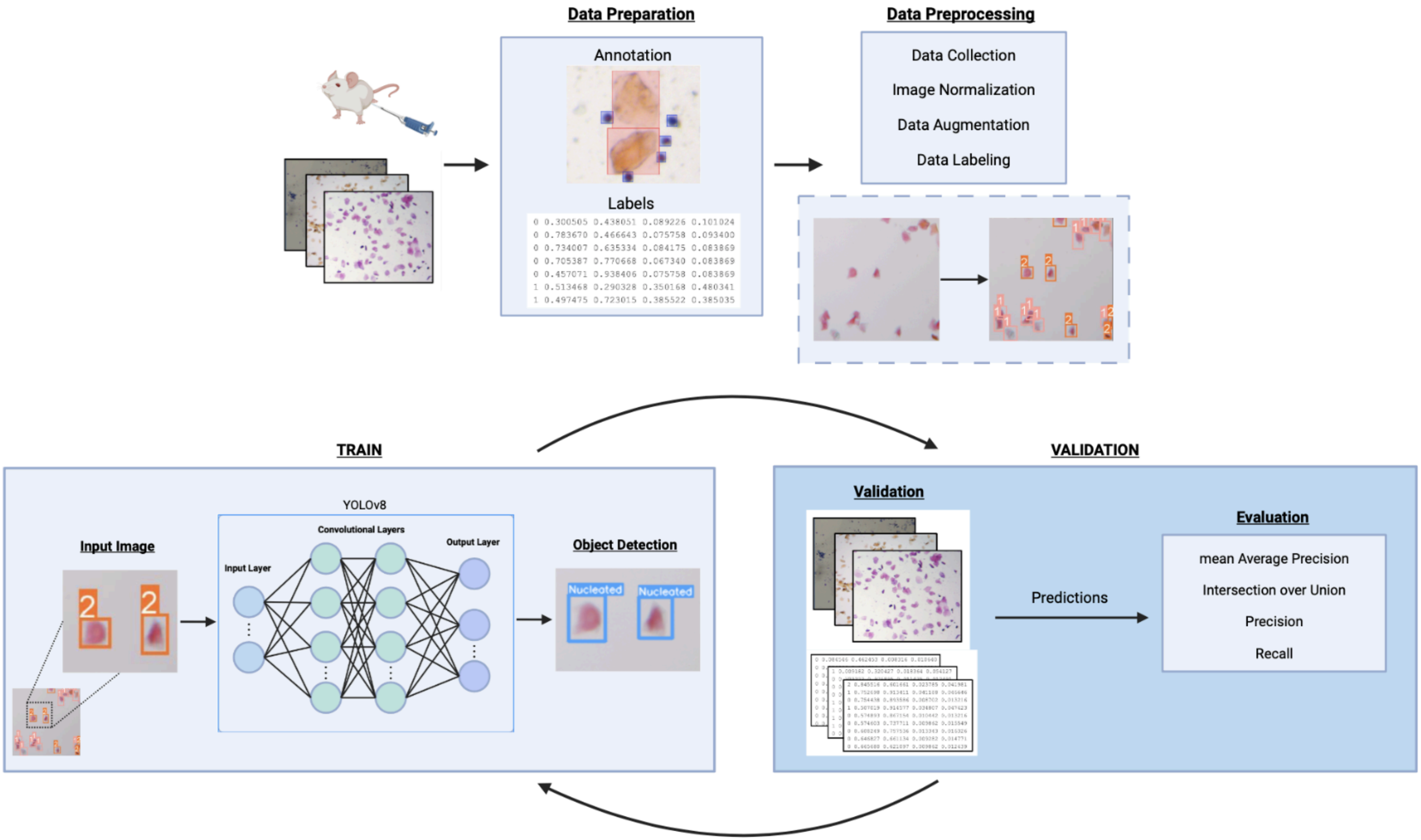

Supplemental Figure 1: Overview of ODES Training. Schematic made with BioRender. Figure 10 illustrates the workflow diagram for supervised machine learning for the estrous cycle images of mice. A select portion of these images were annotated, marking individual cells within the image with their respective labels. The labels for each annotated object were then exported into a format that provides the coordinates for the bounding box (x center coordinate, y center coordinate, width, height). Afterward, the images were organized and placed into Yolov8 for training. The data was first preprocessed via data collection and normalized by image size and color by adjusting the pixel values to a consistent scale. The data was then augmented and labeled to complete the processing and begin the training. The input image is the starting point of the training, where it gets fed into the Yolov8 architecture. This architecture consists of complex machine learning techniques such as convolutional layers for feature extraction, optimization algorithms, and loss functions to adjust the model's parameters. After it runs through the first batch of images and updates its weights, the model validates itself against a separate dataset, the validation dataset. The following metrics are used to evaluate its performance: Mean Average Precision (mAP), Intersection over Union (IoU), Precision, and Recall. IoU is used to measure the precision of the bounding box of the model by comparing the predicted bounding box to the correct bounding box [27]. Precision represents the model's ability to avoid false positives, which is calculated by finding the ratio of true positives and total positive predictions. Recall calculates the ratio of true positives detected and all actual positives, measuring the model's ability to detect all instances of a class. Average Precision (AP) is the area under the precision-recall curve that provides information on the model's precision and recall performance. By extension, mAP calculates the average AP values across multiple object classes. This is useful in multi-class object detection scenarios to provide a comprehensive evaluation of the model's performance. The training and validation sections of the model repeat in a loop for different batches of images until the set number of epochs or until there is no trend of improvement over a set number of epochs (patience).

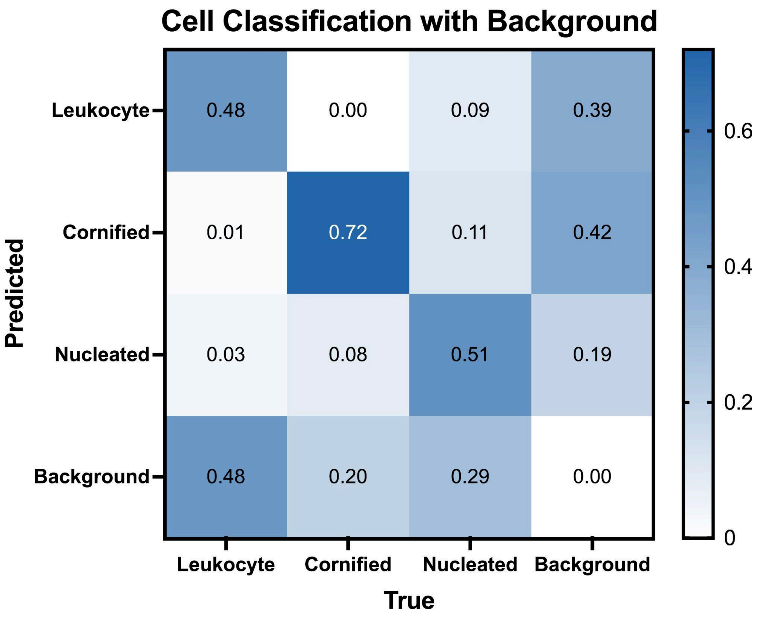

Supplemental Figure 2: Comparative normalized confusion matrices for ODES cell classification with Background. With background classification, leukocytes were correctly identified 48% of the time but misclassified as background in 48% of instances. Cornified cells had a correct classification rate of 72%, with a 20% misclassification rate as the background. Nucleated cells were accurately classified at a rate of 51%, but 29% of instances were mistaken for background and an 8% rate of being mistaken for cornified cells.

a)

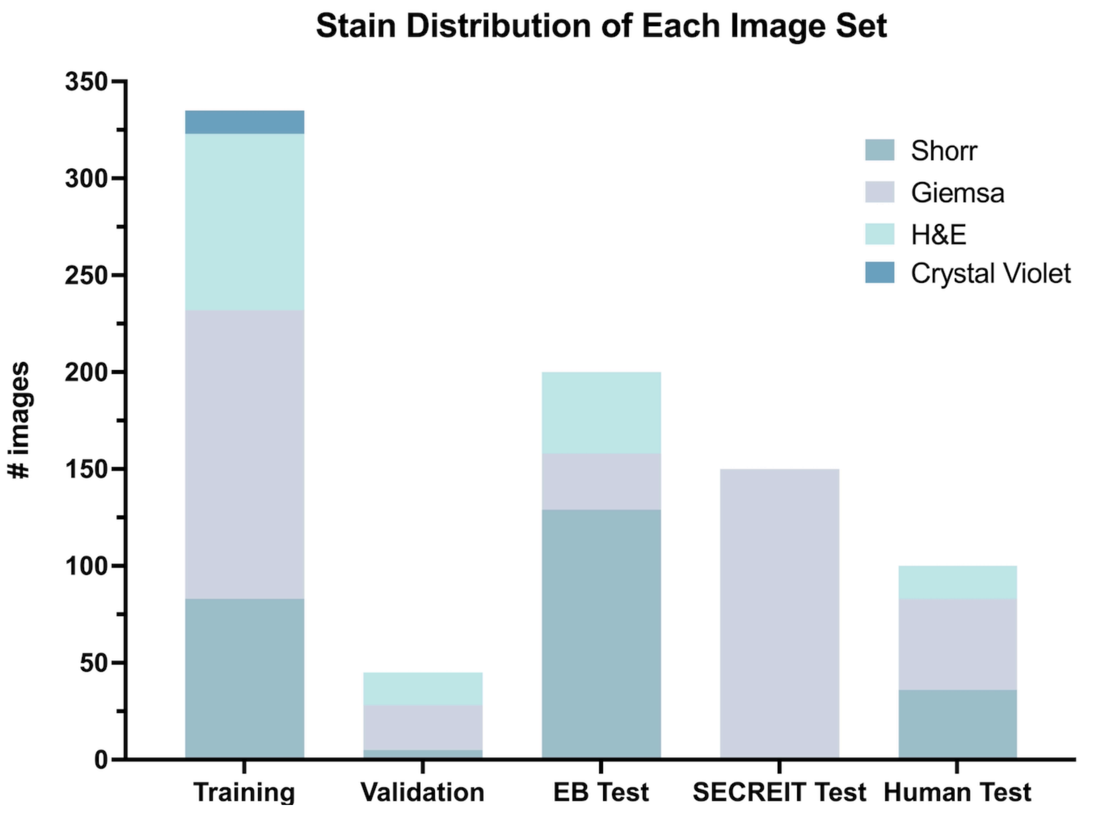
Stain Distribution of Each Image Set
images
350
300
250
200
150
100
50
0
Training
Validation
EB Test
SECREIT Test
Human Test
Shorr
Giemsa
H&E
Crystal Violet

b)

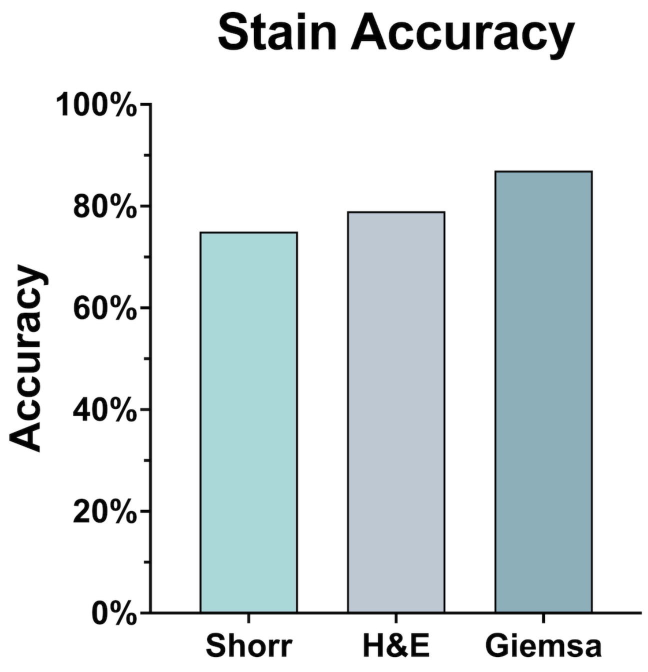
Stain Accuracy
Accuracy
100%
80%
60%
40%
20%
0%
Shorr
H&E
Giemsa

c)

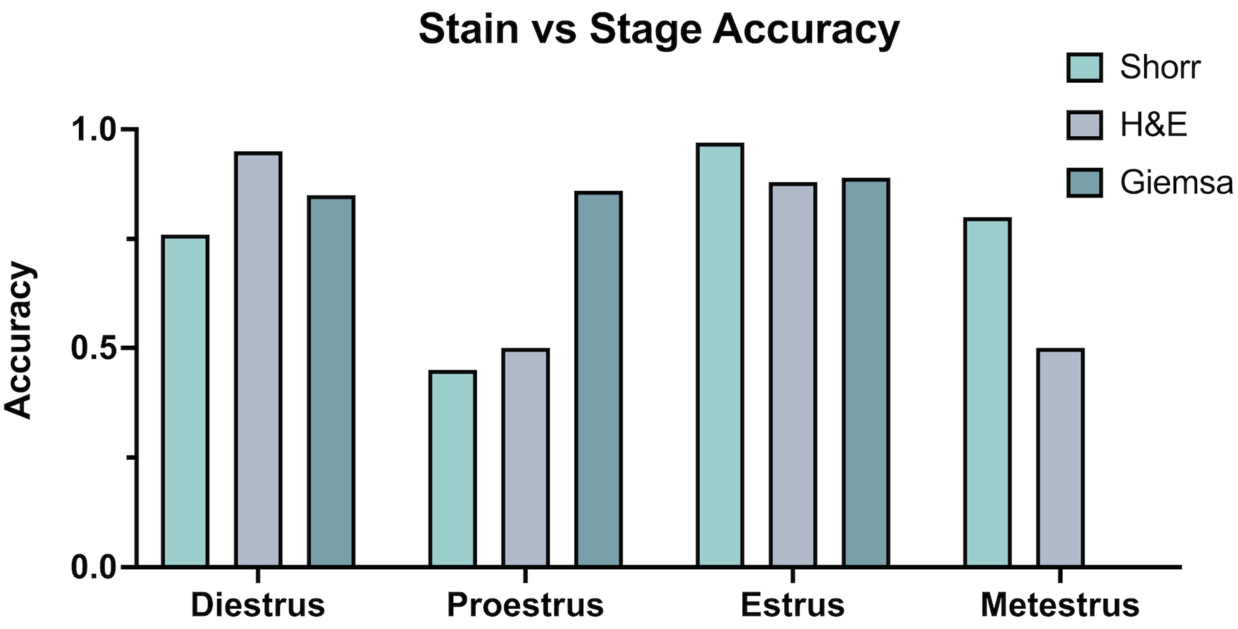

Supplemental Figure 3

a: Distribution of vaginal cytology stains within the training and test sets.

All images in the datasets were randomly placed. The training set included images with all four stains. The EstrousBank dataset predominantly contains images stained with the Shorr stain, supplemented by the Giemsa and H&E stains [11]. The SECREIT test was made exclusively of the Giemsa stained images from the SECREIT paper [10].

b: ODES accuracy for each stain in the test dataset.

ODES performed best on images with a Giemsa stain however not to a degree of statistical significance. This test set included n=129 images with Shorr stain, n=42 images with H&E, and n=179 images with Giemsa divided across the four estrous cycle stages.

c: ODES accuracy for each stain for each stage

For the Diestrus stage, the accuracies are Shorr (76%), Giemsa (85%), and H&E (95%). In the Proestrus stage, the accuracies are Shorr (45%), Giemsa (86%), and H&E (50%). For the Estrus stage, the accuracies are Shorr (97%), Giemsa (89%), and H&E (88%). Lastly, in the Metestrus stage, the accuracies are Shorr (80%) and H&E (50%).There are no significant differences among the stages and stains. No metestrus images were stained with giemsa, therefore this data is excluded.
